# COVID-19 and Global Supply Chain Configuration: Economic and Emissions Impacts of Australia-China Trade Disruptions

**DOI:** 10.3389/fpubh.2021.752481

**Published:** 2021-09-20

**Authors:** Xunpeng Shi, Tsun Se Cheong, Michael Zhou

**Affiliations:** ^1^Australia-China Relations Institute, University of Technology Sydney, Sydney, NSW, Australia; ^2^Department of Economics and Finance, Hang Seng University of Hong Kong, Hong Kong, Hong Kong, SAR China

**Keywords:** COVID 19, supply chain, global value chain, economic integration, Australia, China

## Abstract

Economic shocks from COVID-19, coupled with ongoing US-China tensions, have raised debates around supply chain (or global value chain) organisation, with China at the centre of the storm. However, quantitative studies that consider the global and economy-wide impacts of rerouting supply chains are limited. This study examines the economic and emissions impacts of reorganising supply chains, using Australia-China trade as an example. It augments the Hypothetical Extraction Method by replacing traditional Input-Output analysis with a Computable General Equilibrium analysis. The estimation results demonstrate that in both exports and imports, a trade embargo between Australia and China – despite being compensated for by alternative supply chains—will cause gross domestic production losses and emissions increases for both countries and the world overall. Moreover, even though all other economies gain from the markets left by China, many of them incur overall gross domestic production losses and emission increases. The finding that the Association of Southeast Asian Nations and India may also suffer from an Australia-China trade embargo, despite a gain in trade volume, suggests that no country should add fuel to the fire. The results suggest that countries need to defend a rules-based trading regime and jointly address supply chain challenges.

## Introduction

Ongoing US-China tensions and the economic shocks of the coronavirus disease 2019 (COVID-19) pandemic have raised the attention given to debates around supply chain organisation, with China at the centre of the storm. COVID-19 has changed the world permanently (1) and the supply chain disruption is one of the significant changes. COVID-19 exposed the supply chain vulnerability (2). As part of economic-stimulus packages, many governments have provided incentives to bring home or “reshore” manufacturing. For example, the US government under the Trump Administration led a campaign to exclude China from the trade networks of the United States and its allies (3). Japan is incentivising domestic manufacturing through financial subsidies of ¥240 billion (US$2.3 billion) (4). Australia is working with Japan and India to shift supply chains from China to the Association of Southeast Nations (ASEAN) and India (5). China is also preparing for a more diversified and secure supply chain, motivated not by COVID-19 but by US sanctions on Huawei and other high technology companies. Even for primary resources such as iron ore, China will seek to diversify its supplies (6). The trend towards such political intervention in supply chains, or global value chains (GVC), could undermine the global trade regime and even lead to the collapse of the multilateral trading system (7).

Further understanding the cost and benefits of supply chain manipulation and their distribution among major economies can inform policy development at both national and regional levels. Uncertainties from supply chain configuration will affect emissions directly (8), and indirectly through economic policy uncertainties (9), and thus further affect China's commitments to achieve carbon peak by 2030 and carbon neutrality by 2060 (10). However, quantitative studies that consider both direct and indirect impacts—especially CO_2_ emissions—are limited. The impacts of COVID-19 on supply chains have been extensively examined in the literature. Existing studies on COVID-19 and GVCs mostly focus on the impact of COVID-19 on supply chains (11, 12), the overall measurement of resilience (13, 14), an alternative concept for supply chains (15), prediction of its impact (11) or management of supply chain resilience (16, 17). There has also been discussion of COVID-19's impact on globalisation, albeit mainly in terms of physical restrictions rather than trade (18). More literature have been recorded on the impact of US-China trade disruptions on GVCs (19, 20). However, a recent review found that there is a lack of empirically designed and theoretically grounded studies in COVID-19 related supply chain literature (21). Specifically, there are no studies on how to measure the vulnerability of specific supply chains or the impact of reorganised supply chains on emissions.

The Australia-China trade relationship provides a salient example of supply chain issues. Australia's trade with China has grown rapidly over the last two decades. Goods exports to China grew from A$6.0 billion in 2000 to A$149 billion in 2019, accounting for 38.2 percent of Australia's total goods exports in 2019, while goods imports from China grew from A$9.1 billion to A$79.5 billion over the same period, accounting for 24.7 percent of total imports (22).

**Figure 1:**
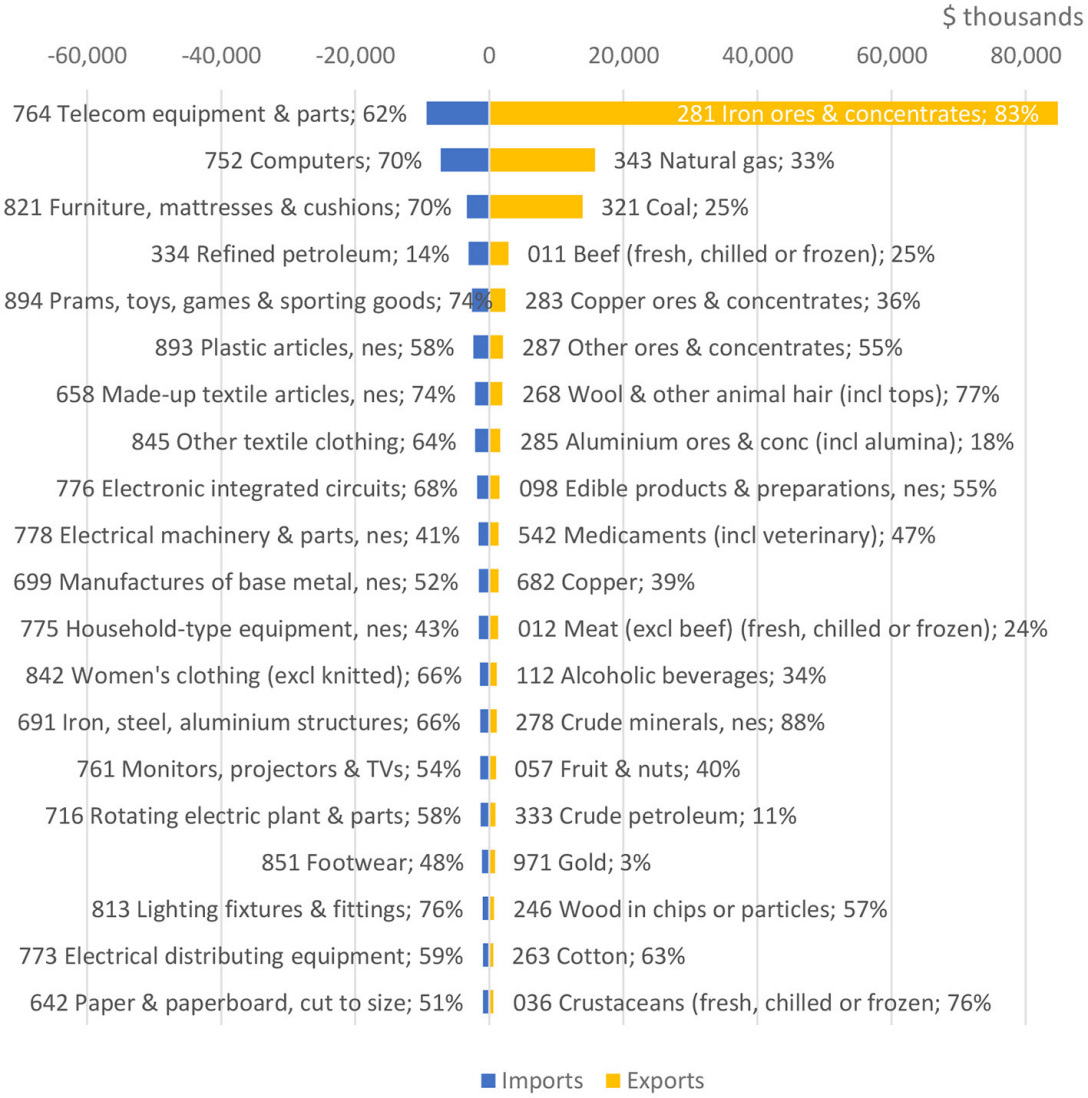
Key trade sectors between Australia and China. %, imports from or exports to CN/total AU imports or exports; f.c.f, “fresh, chilled or frozen; n.e.s, “not elsewhere specified”. Source: Australian Government Department of Foreign Affairs and Trade (2020).

Figure 1 highlights the sectors with the largest trade volume and their respective shares of Australia's total exports and imports per sector. However, the bilateral relationship has deteriorated in the past 2 years and China has since introduced a number of measures restricting Australian exports—including an effective ban on Australia's coal and wine exports to China (23).

Amid increased friction between Australia and China, there have been ongoing debates about whether and where Australia should reduce its dependence on China. There have long been calls for Australia to reroute exports towards markets other than China (24). While some contend that the Chinese restrictions and bans have hurt the Australian economy, others claim the impact has been mitigated by exporting Australia's products to alternative markets (23). Also gaining influence in 2020 were reports of pandemic-induced supply shortages in sectors as wide-ranging as construction, healthcare, agriculture and retail, which prompted an increase in attention given to supply chain organisation and the extent of Australia's dependence on imports (25). The cancellation of the agreements on the Belt and Road Initiative between the state of Victoria and China evidences the tension on the bilateral relationship (26). A comprehensive study can provide timely information to these ongoing debates in both Australia and China.

In this study, we inform global supply chain reorganising debates by examining the case of supply chain disruptions between Australia and China. We first identify the most vulnerable supply chains between Australia and China using the Hypothetical Extraction Method (HEM), an Input-Output (I/O) approach that was recently improved by Duarte et al. (27). Next, we study the impact of alternative supply chains for Australian exports to and imports from China with the global trade and analysis (GTAP) model, a multi-region, multi-sector Computable General Equilibrium (CGE) model that assumes perfect competition and constant returns to scale.

The research makes the following contributions: first, methodologically, we modify the traditional HEM by replacing I/O analysis with a Computable General Equilibrium (CGE) model. Second, this paper is among the first to practically estimate supply chain dependence between Australia and China. Third, we address current debates faced by policymakers by simulating the impact of reorganised supply chains. The policy implications of this case study in Australia-China trade are likely to be applicable to other major trade relationships between China and the developed world.

## Methodology

The research ranks Australia's exports to China and imports from China by sector. In determining the importance of each sector, we adopt the HEM, which measures the importance of each sector by simulating the impact of an export or import embargo in that sector. The simulations are conducted using a CGE model, which estimates the economic and emissions consequences of shutting down bilateral trade for each sector. The sectors are ranked in descending order based on the magnitude of economic loss. We then report the economy-wide impact for selected economies/economy groups after removing each of their five top-ranked sectors. Australia's gas export is simulated separately as Australia now is the world largest liquid natural gas (LNG) exporter, and a significant proportion goes to China (28).

### Hypothetical Extraction Method (HEM)

In most studies, the importance of a sector in trade is assessed using final imports as a measurement of interdependence. However, this approach is insufficient as 70 percent of global trade is in intermediate goods (29), meaning that finished goods may have supply chains beyond where they are assembled.

The importance of each sector in bilateral imports and exports is more appropriately be ranked by the HEM. The HEM is an application of an Input-Output (I/O) approach that was first initiated by Schultz (30) and further improved by Cella (31) and Duarte at al (27). In recent years, the HEM was extended to identify the key sectors in terms of emissions through case studies of Beijing (32), China (33), and South Africa (34).

The HEM measures the importance of a sector in an economy by estimating the economic loss when that sector is hypothetically extracted from the economy. The difference in GDP between this hypothetical scenario and the baseline scenario is the economic effect or “value” of this sector. By estimating the values one by one, we get a ranking for Australia's import/export from China. The basic concept of the HEM is shown by Huang and Tian (35).

Traditionally, the HEM estimation is concluded in an I/O model. However, the I/O model has several limitations, such as a lack of constraints in the supply side and budget for households and governments, fixed prices in the model with no consumer response to price changes, and also, the ratios for intermediate inputs and outputs are fixed in the model. These assumptions are therefore unreasonable and could lead to misleading results.

### The Computable General Equilibrium (CGE) Model

In this paper, we replace traditional I/O analysis with the GTAP model. The GTAP Model is a multi-region, multi-sector CGE model which assumes perfect competition and constant returns to scale. We use the current model (v7) (36) with the most updated database (Version 10) that is based on the world economy in 2014 (37). It is worth noting that this study is based on an investigation of the likely outcomes of the different scenarios. Given that the database is comprised of data available in 2014, caution should be exercised in interpreting the results. Hence it is suggested that readers should not place too much emphasis on exact numerical values derived from each scenario, but should instead focus on relative impacts and their associated consequences in each scenario. Compared with the original I/O analysis in the HEM framework, the CGE model can better reflect reality.

The GTAP database is a global database describing bilateral trade patterns, production, consumption and intermediate use of commodities and services for 65 sectors and 141 countries and regions in the world with millions of data. The GTAP database and its associated CGE models are very comprehensive as they include all components of an economy and allow these components to interact with each other. In addition, the model describes the efficiency-maximising behaviour of firms and the utility-maximising behaviour of consumers. Because of the comprehensiveness and the accuracy of the GTAP database and its CGE model, they are widely employed by researchers around the world in conducting CGE analysis and policy simulation.

Shocks in the form of changes in policy variables can be applied to the model for policy simulation. Results can then be derived by working out new equilibrium values for all variables. Since all components in an economy are linked together in the model, one can observe the impacts of the shocks on all the sectors and also evaluate the effects on GDP, employment, price, supply, and demand in all sectors. The GTAP model is deemed the most advanced tool in CGE analysis on international trade; therefore, it is employed in this study.

In the database, there are 65 sectors, as shown in the Appendix. Since our interest is to rank the importance of each sector, we do not aggregate the sectors. Among the 65 sectors, one sector is “Dwellings” that does not involve trade, and thus we ignore this sector. Based on the ranking of the 65 sectors in import and export, respectively, we identify the key sectors from Australia's perspective.

Regions, however, are aggregated into 14 regions. Apart from Australia and China, other independent regions include Hong Kong and the US. Hong Kong is separated because some trade from China might be rerouted through it. The US is separated because of its size and ongoing tension with China. ASEAN and India are highlighted as they are likely to be the destination for trade relocated from China. The European Union is separated as they are likely to have different policies from East and Central Europe (Table 1).

**Table 1:** Regional groups.

**Region**	**Economy or economic group**
Asia (6)	ASEAN, India, China, Hong Kong, Developed Asia (Japan, Korea, Taiwan), Developing Asia
The Pacific (1)	Australia
North America (2)	US, Other North America
Africa (1)	Africa
Central-South America (1)	Central-South America
Europe (2)	European Union, Other European economies
Others (1)	Rest of the world

In order to verify that the results are consistent, similar scenarios have been run on previous databases of 2007 and 2011. Very similar results have been obtained, and all key conclusions are robust. However, for the sake of brevity, only simulation results derived from the latest database are presented, while results from previous databases can be provided upon request.

### Economic and Environmental Impacts of Reducing Australia's Trade With China

We use GDP change (measured in USD unless otherwise stated) as the key indicator to separately rank Australia's exports to China and imports from China by sector. The ranking provides an indicator of the importance of each sector in bilateral trade. Our estimation suggests that a total trade embargo between Australia and China will cause economic losses and increased carbon emissions for both countries and the world as a whole—though some economies may benefit from the trade cut. Although part of the reduction in bilateral trade can be diversified to other regions, the trade that has been ceased cannot be fully compensated for, and costs to both countries and the global communities remain significant. Furthermore, although a significant number of economies can increase their share in Australia's market after China is excluded, many of these economies may still register a loss in GDP. This is because China is the world's second-largest economy: if China suffers an economic loss, consumption in China declines, which may lead to a reduction in imports from other economies.

### Impact of Cutting Off Australia's Exports to China

**Figure 2:**
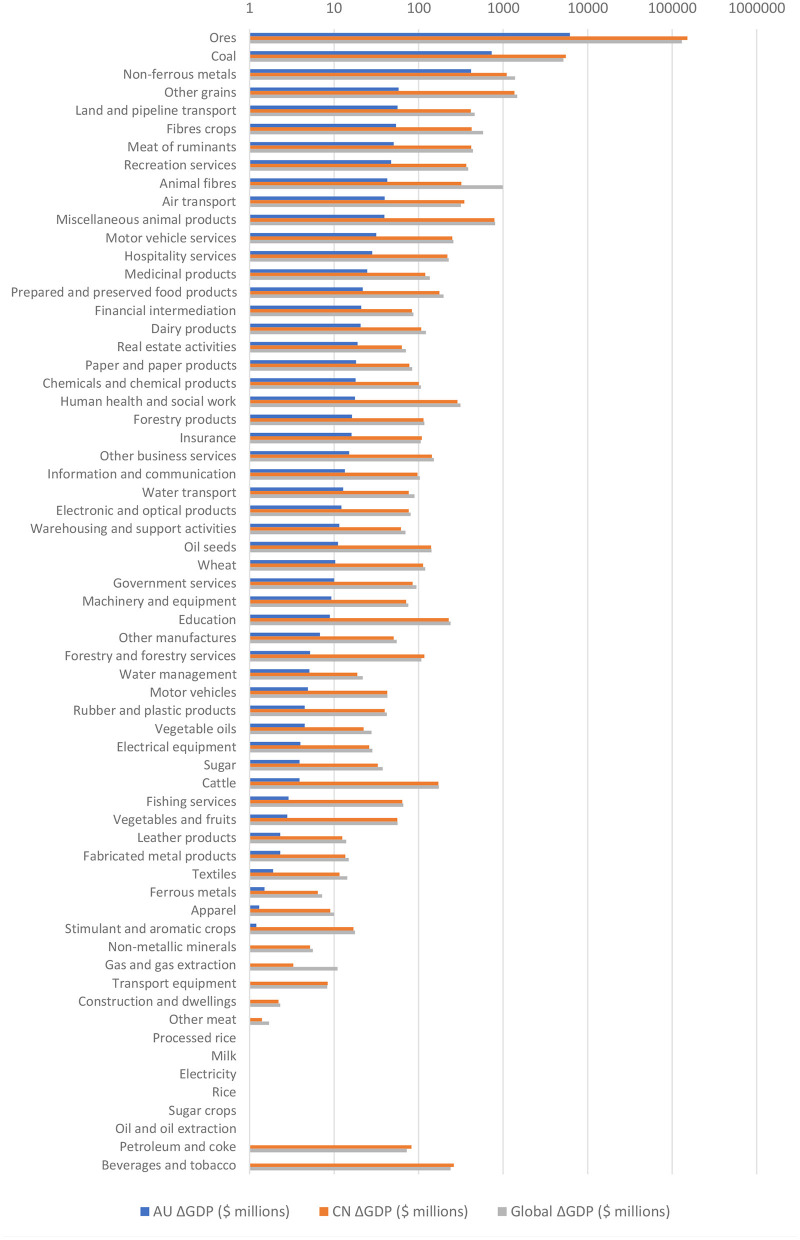
Loss in GDP from trade cut-off per sector, Australia exports to China (M$). The x-axis is in logarithmic scale; in the case of loss than $1 million, or gains (for the two cases oil & oil extraction and berate & tobacco), the values are not shown in the figure. Source: Authors' own estimation.

Figure 2 illustrates loss in GDP from the cessation of Australian exports to China in each sector. These are ranked by the sizes of the loss in Australia's GDP resulting.

As expected, the largest GDP losses for Australia are incurred when the largest goods export sectors are cut off. For example, cutting off exports of Ores—which includes Australia's largest export good, iron ore—results in a GDP reduction of $6,200 million. However, the absence of this trade causes a $150,000 million in China's GDP, 24.5 times larger than the GDP loss for Australia.

At the aggregate global level, cutting off trade in the top four sectors—Ores, Coal, Non-ferrous metals and Other grains—each causes a net GDP loss of over $1,000 million. Cutting off Ores causes a net $130,000 million loss globally, several multiples larger than the next largest sector, Coal, which results in a $5,200 million loss.

In 61 of 63 sectors, China's GDP contracts more than Australia's when trade is disrupted. However, given that China's economy was 10 times of the Australian economy, the relative impact to China may be much smaller than to Australia's in many sectors. At the global level, blocking trade in any sector would cause a net GDP contraction.

When it comes to the environmental impacts of cutting off trade, Table 2 shows that emissions changes are again concentrated in Australia's mineral exports to China. As with changes in GDP, Australia experiences a larger emissions reduction than China in most of the sectors. Most notably, cutting off Ores exports would reduce China's emissions by 123.8 MT. Cutting off Coal, however, causes an emissions increase in both economies, but the emissions increase would be much larger in China than in Australia. And cutting off Australian exports of Non-ferrous metals results in a 3.1 MT emissions reduction in Australia, while China would see a small increase of 0.6 MT.

**Table 2:** Change in emissions from trade cut-off per sector, Australia exports to China (Mt).

	**Australia**	**China**	**World total**
Ores	12.8	−123.8	−24.7
Coal	4	54.2	73.2
Non-ferrous metals	−3.1	0.6	−0.2
Other grains	−0.2	−0.2	0.2
Land and pipeline transport	−0.3	−0.2	0
Fibres crops	−0.4	−0.1	0.2
Meat of ruminants	−0.1	−0.2	−0.1
Recreation services	0	−0.3	0
Animal fibres	−0.3	−0.2	−0.1
Air transport	−0.9	0.1	0.1
Miscellaneous animal products	−0.1	−0.2	0
Motor vehicle services	−0.1	−0.4	−0.1
Hospitality services	0	−0.2	−0.1
Real estate activities	0	−0.1	0
Paper and paper products	0	0	0.1
Chemicals and chemical products	−0.1	0	0.1
Human health and social work	0	−0.1	0
Forestry products	−0.1	−0.1	−0.1
Insurance	0	−0.1	0
Other business services	0	−0.2	−0.1
Information and communication	0	−0.1	0
Water transport	−0.1	0	0
Warehousing and support activities	0	−0.1	0
Education	0	−0.1	0
Forestry and forestry services	−0.1	−0.1	−0.2
Cattle	0	−0.1	−0.1
Petroleum and coke products	0	−0.1	−0.1
Beverages and tobacco products	0	−0.1	0

*Sectors absent have no change for Australia, China and the World Total*.

*Source: Authors' own estimation*.

Cutting off exports in other sectors would cause somewhat less significant changes in emissions, with the exception of Air transport, which would lead to a 0.9 MT emissions reduction in Australia but a 0.1 MT increase in China.

At the global level, cutting off Ores results in a net emissions reduction of 24.7 MT, but this comes at the cost of $130.1 billion in lost GDP. Cutting off Coal, however, causes a net emissions increase of 73.2 MT globally, in addition to the cost of a $51.6 billion contraction in worldwide GDP. For other sectors, the emissions impact is much lower, with a maximum emissions change of negligible at 0.2 MT.

### Impact of Cutting Off Australia's Imports From China

**Figure 3:**
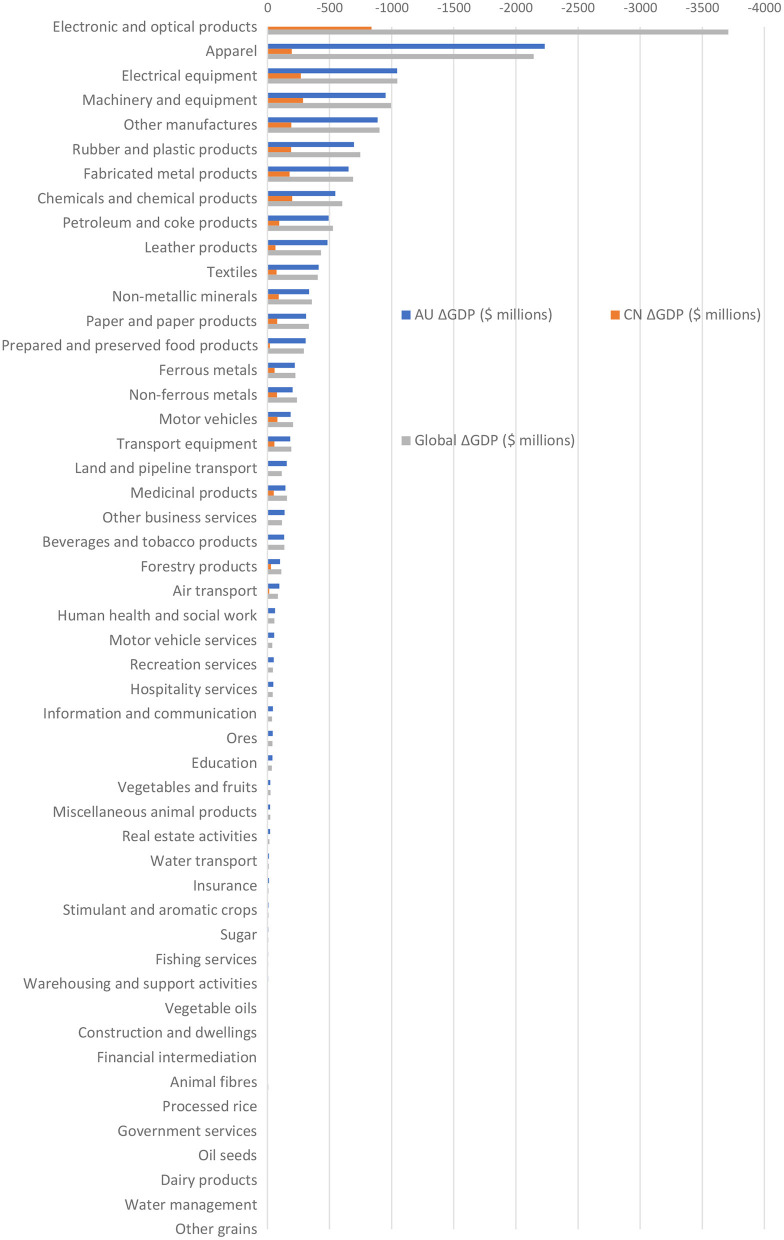
Change of GDP due to trade cut-off by sector, Australia's imports from China, M$. Thirteen sectors with change of GPD between 0 to 0.9 million $ are not included. Source: Authors' own estimation.

Figure 3 illustrates the effect of cutting off the flow of imports from China to Australia on GDP and emissions in Australia, China, and the world.

In contrast to Figure 2, there are no sectors in which Australia sees increased GDP following trade cessation. In three sectors Australian GDP decreases by more than $1,000 million: Electronic and optical products, by $3,500 million; Apparel, by $2,200 million; and Electrical equipment, by $1,000 million. These Manufactures correspond to the largest flows of products from China to Australia.

In 60 of 63 sectors, Australian GDP contracts by more than Chinese GDP. In three sectors—Apparel, Electronic and optical products, and Electrical equipment – the GDP losses for China are 11.4, 4.2, and 3.9 times smaller than the GDP loss for Australia, respectively. There are also a number of sectors where China sees GDP increases following stopped trade, most notably including Land and pipeline transport, where China gains $7.7 million while Australia loses $155.5 million.

GDP changes at the global level generally exceed that of Australia's. In the top three sectors, the global GDP loss is $194.7 million larger, $90.4 million smaller and $1.5 million larger than the Australian GDP loss in Electronic and optical products, Apparel and Electrical equipment, respectively.

The emissions impact of stopping Chinese exports to Australia is also considerably less significant than that of stopping Australian exports to China (Table 3). Only 26 of the 65 sectoral cases record at least one non-zero impact. No sector accounts for an emissions change >1 Mt in either Australia or China, with the majority of sectors recording negligible emissions changes of <0.05 Mt. The maximum of a 1 Mt reduction in Australia comes from stopping Chinese exports of Petroleum and coke products. This corresponds with an emissions increase of 0.1 Mt in China.

**Table 3:** Change in emissions from trade cut-off per sector, China exports to Australia (Mt).

	**Australia**	**China**	**World total**
Electronic and optical products	−0.5	0.7	1.9
Apparel	−0.1	0.3	0.3
Electrical equipment	−0.2	−0.2	0.1
Machinery and equipment	−0.1	−0.1	0.4
Other manufactures	0	0.2	0.4
Rubber and plastic products	0	−0.2	0
Fabricated metal products	0	−0.3	−0.1
Chemicals and chemical products	0	−0.9	−0.8
Petroleum and coke products	−1	0.1	−1.1
Leather products	−0.1	0.1	0.2
Textiles	0	−0.1	0
Non-metallic minerals	0.2	−0.8	−0.3
Paper and paper products	0	−0.1	0
Prepared and preserved food products	0	0.1	0.1
Ferrous metals	0	−0.5	−0.1
Non-ferrous metals	0	−0.3	−0.1
Motor vehicles	0	0	0.1
Transport equipment	0	0	0.1
Land and pipeline transport	0.1	0	0.2
Medicinal products	0	0.1	0.1
Other business services	0	0.1	0.1
Air transport	0.1	−0.1	0.1
Motor vehicle services	0	0.1	0.1
Ores	−0.1	0	0
Rice	−0.6	0	−0.6
Gas and gas extraction	0	0.1	0.1

*Sector recording zero impact for all three indicators are not included*.

*Source: Authors' own estimation*.

In a number of other sectors, such as Electronic and optical products and Apparel, cutting off Chinese imports to Australia also results in significantly larger emission increases in China than in Australia. An opposite effect is observed in Non-metallic minerals, where Australian emissions increase by 0.2 Mt while Chinese emissions decrease by 0.8 Mt.

Taking Table 2 into account, Australia is more likely to see emissions benefits of cutting off trade with China in both imports and exports. Such an emissions reduction, however, is not an unqualified positive since the associated decline in GDP is also substantial.

At the global level, cutting off the Australia-China trade also results in net emissions changes lower than 0.05 Mt for most sectors. The largest emissions change comes in Electronic and optical products, with a 1.9 Mt increase.

## Regional Economic and Environmental Impacts of Alternative Markets and Suppliers For Australia

In this section, we investigate the economic and environmental changes in different economies/economy groups after key Australian exports to and imports from China are disrupted.

### Regional Economic and Emission Impacts of Cutting Off Australia's Exports to China

Table 4 presents regional GDP and CO_2_ emissions impacts of cutting Australian exports to China in key sectors. Column (1) lists the regions, while columns (2) to (7) list the GDP change per region following the cessation of Australia's exports to China in each of the six key sectors discussed in Figure 2. Negative values indicate GDP losses, while positive values indicate GDP gains. Columns (8) to (13) list the CO_2_ emissions changes following trade cessation in the same six sectors. Negative values indicate decreased CO_2_ emissions, while positive values indicate increased CO_2_ emissions.

**Table 4:** Economic and emissions impact of cutting off Australian exports to China in key sectors.

	**ΔGDP ($ millions)**						**ΔCO_2_ (Mt)**					
	**Other grains**	**Meat of ruminants**	**Animal fibres**	**Coal**	**Gas and gas extraction**	**Ores**	**Other grains**	**Meat of ruminants**	**Animal fibres**	**Coal**	**Gas and gas extraction**	**Ores**
Australia	−58.0	−50.8	−42.5	−732.6	−0.5	−6173.9	−0.2	−0.1	−0.3	4.0	0.0	12.8
China	−1358.6	−419.0	−319.8	−5522.6	−3.3	−151296.0	−0.2	−0.2	−0.2	54.2	0.0	−123.8
Hong Kong	−0.1	0.0	0.0	0.2	0.0	−7.9	0.0	0.0	0.0	0.0	0.0	0.7
ASEAN	−3.5	−0.8	−4.5	11.8	−0.2	305.0	0.0	0.0	0.0	−1.3	0.0	3.2
Developed Asia	−1.8	32.4	−10.7	644.1	−0.1	2843.3	0.1	0.1	0.1	12.3	0.0	16.3
India	−5.7	−0.3	−9.0	84.4	0.0	−341.9	0.1	0.0	0.1	5.3	0.0	7.4
Developing Asia	−0.4	−0.1	−0.3	−0.5	0.0	6.8	0.0	0.0	0.0	0.0	0.0	0.1
US	10.1	−11.3	−12.7	30.7	0.0	4305.6	0.3	0.0	0.0	1.3	0.0	8.6
Other North America	−2.3	−2.6	−1.8	11.0	0.0	1584.7	0.0	0.0	0.0	−0.1	0.0	2.5
Central- South America	−3.3	9.4	8.6	6.3	−0.1	5295.1	0.0	0.0	0.1	0.4	0.0	14.1
EU + UK	−32.4	4.6	−596.4	324.5	−0.6	7291.7	0.1	0.0	−0.1	1.6	0.0	3.0
Non-EU Europe	−7.0	−3.0	−15.5	−13.4	−6.9	1640.6	0.0	0.0	0.0	−0.3	0.0	7.9
Africa	−1.2	2.0	11.9	−15.3	0.1	2695.2	0.0	0.0	0.1	0.1	0.0	14.5
Rest of world	−4.6	−1.5	2.8	9.0	0.6	1736.1	0.0	0.1	0.1	−4.3	0.0	8.1
World total	−1468.9	−440.9	−990.0	−5162.5	−11.0	−130115.7	0.2	−0.1	−0.1	73.2	0.0	−24.7

Columns (2) to (7) show that the GDP impacts for economies other than Australia and China are mixed. For instance, ASEAN's GDP declines in four of six sectors when Australian exports to China are cut off. India, meanwhile, also sees GDP losses in four of six sectors. This suggests that current efforts to relocate Australia's supply chains from China to India could be counter-productive. Where there are GDP gains, the largest tend to be in established markets such as Developed Asia, the US or the EU and UK.

Consistent with Figure 2, the GDP impacts of stopping Australian exports of Ores to China are the largest among the six sectors. Column (7) indicates that aside from Australia and China, Hong Kong and India both lose $7.9 million and $341.9 million in GDP, respectively. Ten of 14 regions experience GDP gains, including ASEAN ($305.0 million), but these are vastly outweighed by China's $151 billion GDP loss, which on its own causes world GDP to fall by a net $130 billion. The largest GDP gains are made by the EU and UK ($7.3 billion), Central and South America ($5.3 billion) and the US ($4.3 billion).

Column (5) lists the GDP changes associated with cutting off Australian Coal exports to China. Here, nine of 14 regions see GDP growth, including ASEAN ($11.8 million) and India ($84.4 million). However, these are significantly smaller than GDP gains made by Developed Asia and the EU and UK, which respectively see GDP growth of $644.1 million and $324.5 million. This is because Developed Asia is the main market for Australian coal and thus benefits from lower prices due to the absence of Chinese demand.

In Other grains [column (1)], cutting off Australia's exports to China induces GDP contractions in all regions except the US, which grows its GDP by $10.1 million. ASEAN loses $3.5 million in GDP, while India loses $5.7 million. Similarly, in Animal fibres, the GDP impact tends to be negative for most regions, including ASEAN and India.

For Meat of ruminants, Developed Asia, Central and South America, the EU and UK and Africa see GDP gains of $32.4 million, $9.4 million, $4.6 million and $2.0 million, respectively. Hong Kong's GDP experiences no change, with the remaining regions all declining in GDP.

In terms of emissions, disrupting Australian exports to China has particularly significant effects in the case of Ores and Coal. Column (13) shows that while China's emissions decrease by 123.8 MT, all other regions' emissions increase by 0.1 MT (Developing Asia) to 16.3 MT (Developed Asia). The latter emissions change is the second largest after China's.

In the Coal sector, all regions experience emissions increases, with the exception of ASEAN, Other North America, non-EU European economies and the rest of the world. Outside China, the only region with an emissions change in the double digits is Developed Asia.

In other sectors, no regions outside Australia and China experience emissions changes >0.25 MT, except for the US, which increases its emissions by 0.3 MT when Australian exports of Other grains are redirected. The overall emissions impact in these key sectors is therefore dominated by the emissions-increasing effects of cutting off Australia-China trade in Ores and Coal.

### Regional Economic and Environmental Impacts of Cutting Off Australia's Supply Chains From China

Similar to Tables 4, 5 presents the GDP and CO_2_ emissions impacts when Australia's imports from China are cutting off in each of the five key sectors. Column (1) lists the regions, and columns (2) to (6) list the GDP changes for each region in each of the five key sectors in Figure 3. Positive values denote GDP increases, while negative denotes GDP decreases. Columns (7) to (11) list the CO_2_ emissions changes for each region in the five sectors, with positive values indicating increased emissions and negative values indicating decreased emissions.

**Table 5:** Economic and emissions impact of cutting off Australian imports from China in key sectors.

	**ΔGDP ($ millions)**					**ΔCO_2_ (Mt)**				
	**Apparel**	**Medicinal products**	**Electronic and optical products**	**Electrical equipment**	**Machinery and equipment**	**Apparel**	**Medicinal products**	**Electronic and optical products**	**Electrical equipment**	**Machinery and equipment**
Australia	−2232.9	−144.1	−3516.4	−1044.4	−950.4	−0.1	0.0	−0.5	−0.2	−0.1
China	−196.4	−50.0	−837.3	−269.0	−286.7	0.3	0.1	0.7	−0.2	−0.1
Hong Kong	0.0	0.0	−0.1	−0.1	0.0	0.0	0.0	0.0	0.0	0.0
ASEAN	35.5	−0.4	40.8	11.7	12.8	0.1	0.0	0.3	0.1	0.1
Developed Asia	13.9	−1.2	71.1	29.1	30.1	−0.1	0.0	0.4	0.1	0.1
India	16.7	1.8	4.2	11.3	6.6	0.0	0.0	0.2	0.0	0.1
Developing Asia	34.0	0.1	1.1	0.7	0.5	0.0	0.0	0.0	0.0	0.0
US	−7.1	10.8	201.8	45.6	42.2	0.0	0.0	0.6	0.1	0.2
Other North America	14.4	1.1	39.1	11.5	9.5	0.0	0.0	0.1	0.0	0.0
Central and South America	15.6	−0.8	21.4	7.9	8.7	0.0	0.0	0.0	0.0	0.0
EU + UK	56.7	25.9	200.0	113.6	105.6	0.0	0.0	0.1	0.1	0.1
Non-EU Europe	15.1	−4.8	1.4	0.1	0.4	0.0	0.0	0.0	0.0	0.0
Africa	34.6	1.9	26.2	12.6	10.5	0.0	0.0	0.0	0.0	0.0
Rest of world	57.5	2.3	35.8	23.5	15.9	0.1	0.0	0.1	0.0	0.0
World total	−2142.5	−157.4	−3711.1	−1045.9	−994.3	0.3	0.1	1.9	0.1	0.4

In Table 5, a majority of regions (excepting Australia and China) see GDP gains after Australia-China supply chains are severed in each sector. In particular, ASEAN gains in four out of five sectors, while India gains in all five. Overall, however, India's GDP gains tend to be smaller than that of ASEAN or other, more established suppliers.

As expected, GDP tends to increase in the regions from which Australia imports a larger proportion of the total in each sector after blocking Chinese exporters. For instance, after cutting off Australia's imports of Electronic and optical goods from China, the absolute value of US exports in this sector to Australia grows by $3.5 billion and its GDP increases by $201.8 million [column (4), Table 5]. Similarly, the EU and UK's combined GDP increases by $113.6 million when it boosts its exports of Electrical equipment to Australia by $1.6 billion. And overall, no region's absolute GDP change exceeds that of Australia or China.

However, there are exceptions to this pattern. Despite growing its exports of Apparel to Australia by $265.6 million, the US' GDP contracts by $7.1 million overall. This phenomenon is particularly pronounced in the Medicinal products sector, with four out of 14 regions registering a similar result. In particular, whereas India boosts both its GDP and exports to Australia (by $1.8 million and $16.9 million respectively), ASEAN's GDP falls by $0.4 million, despite increasing sectoral exports by $25.8 million. Similarly, non-EU European economies' collective GDP declines by $4.8 million, even as exports of Medicinal products increase by $120.1 million.

Columns (2) to (6) also indicate that the US and the EU (with the UK) regions tend to experience the largest GDP gains from Australia after redirecting its supply chains away from China. In some sectors, this occurs even if they do not become the largest exporters in those sectors or have the largest increase in the absolute value of their exports in that sector. For example, in Electronic and optical goods, the US and the EU (with the UK) regions increase the absolute value of their exports to Australia by $3.5 billion and $3.1 billion, respectively. These increases come in second and third place to ASEAN's $4.5 billion export growth in the same sector, but this is not reflected in changes to the regions' GDP. The US' GDP expands by $201.8 million, and the EU and UK's expands by $200.0 million, but ASEAN's expands by a significantly smaller $40.8 million.

In terms of emissions impacts, blocking Australia-China supply chains in these sectors tends to have relatively small effects. The largest emissions change outside China of a 0.6 MT increase occurs in the US when Australia's Electronic and optical goods imports from China are cut off, followed by a 0.4 MT increase in Developed Asia in the same sector. The total global emissions increase in this scenario is 1.9 MT.

## Conclusion and Policy Implications

The economic shocks of COVID-19, coupled with ongoing US-China tensions, have reinvigourated debates around supply chain organisation with China as the focal point of discussion. Many analysts and even governments are actively promoting the relocation of supply chains away from China. Such interventions in supply chains could undermine the global trade regime and economic integration, particularly in East Asia, which has significantly benefited from integrated regional production networks. While advocates are optimistic about reorganising supply chains, the complexity of global value chains and integration of the global economy suggests that the direct and indirect impacts may be unexpected. However, comprehensive studies of alternative global value chains are limited.

In this study, we examine debates on supply chain organisation and the economic and emissions impact of rerouting supply chains using Australia and China trade as an example. Australia-China trade is a useful case study as both countries have complementary advantages in trade but are engaged in trade tensions. We first identify the most vulnerable supply chains between Australia and China. We augment the Hypothetical Extraction Method by replacing traditional Input-Output analysis with a Computable General Equilibrium analysis. We selected gross domestic output and emission changes as the key indicators in our analysis and separately rank Australia's exports to China and imports from China to Australia according to gross domestic product change sector by sector. The ranking provides an indication of the importance of each sector in bilateral trade. We also study the regional economic and emissions impacts of supply chain reorganisation in key Australia-China trade sectors when these trade sectors are forced to be cut off individually.

The estimation results demonstrate that in both exports and imports, a trade disruption between Australia and China—despite being compensated for by alternative supply chains—will cause economic losses and emissions increases for both countries and the world total. Losses are diversified across sectors due to factors such as the size and substitution of the affected trade.

Further analysis of the regional economic and emissions impact after Australia-China trade decoupling in six key Australian export sectors and five key import sectors found that although all other economies gain in the markets left by China, many of them suffer from overall economic losses and emissions increases. The impact on trade flows is more significant when disrupting Australia's exports than its imports. A total trade cut of Australia's exports to China by sector could result in a reduction of Australia's exports in each of these sectors by about 40 percent. This suggests that no other economy can fully replace the Chinese market for Australian exports. In contrast, in the case of Australia's imports from China, severed trade between Australia and China does not induce a significant change in Australia's imports, which suggests that Australia's imports from China can be substituted, albeit with potentially increased prices. Interestingly, ASEAN and India, which are expected to benefit from Australia's decoupling from China, respectively lose in four and five out of the total six sectoral cases.

Our study generates the following implications:

First, despite the vulnerabilities of global supply chains revealed by COVID-19, deglobalisation is not a rational solution. This is supported by the results of our modelling which demonstrates that reducing bilateral trade causes significant economic and environmental losses for both countries and the world. Since Australia and China have a complementary economic structure, and because trade between them can deliver significant economic and emissions benefits for both countries and the world, political interventions should be avoided.

Second, *ad hoc* diversification of imports or exports may not be a rational solution. Our simulations suggest that while some economies may benefit from supply chain diversification, a forced reorganisation will work against world trade dynamics—thus incurring economic and environmental costs and meaning that there is a dead-weight loss for the global community.

Third, regional integration should be upheld rather than undermined. While the pandemic reveals the vulnerability of global supply chains, it also shows that supply chains are resilient. Supply disruptions in India in the first half of 2021 indicates that zero-sum supply chain rerouting or replacement may not be an adequate solution.

Last, a collaborative approach in preparing for pandemics and other disasters is desirable. The pandemic is a global challenge, requiring collective efforts. Problems with supply chains can be mitigated by sharing of technologies and production capacity. For example, many countries lack the domestic capacity to produce vaccines, and thus global coordination in vaccination distribution is crucial in preventing the spread of the pandemic.

One caveat of our study is the reference year does not fully reflect the current situation. The current version of the GTAP model uses the global economic system in 2014 as the reference. Although it can predict the relative change and direction for various policy scenarios, it cannot represent the current situation in absolute terms. For example, since Australia's large proportion of LNG exports only started in 2015, our estimation by sector does not capture the importance of gas trade. Future studies could further calibrate the key indicators to the year 2019 for more precise estimations.

## Data Availability Statement

The data analysed in this study is subject to the following licences/restrictions: It can be purchased from the Centre for Global Trade Analysis at the Purdue University. Requests to access these datasets should be directed to https://www.gtap.agecon.purdue.edu/databases/default.asp.

## Author Contributions

XS: conceptualisation, methodology, visualisation, and writing. TC: conceptualisation, methodology, software, analysis, and reviewing. MZ: writing, validation, and editing. All authors contributed to the article and approved the submitted version.

## Funding

This research was funded by the Economic Research Institute for ASEAN and East Asia (ERIA) and National Natural Science Foundation of China (#71828401 and 72174056).

## Conflict of Interest

The authors declare that the research was conducted in the absence of any commercial or financial relationships that could be construed as a potential conflict of interest.

## Publisher's Note

All claims expressed in this article are solely those of the authors and do not necessarily represent those of their affiliated organizations, or those of the publisher, the editors and the reviewers. Any product that may be evaluated in this article, or claim that may be made by its manufacturer, is not guaranteed or endorsed by the publisher.
