# Supplementary material for: COVID-19 and Global Supply Chain Configuration: Economic and Emissions Impacts of Australia-China Trade Disruptions
Source: Front Public Health. 2021 Sep 20;9:752481. doi: 10.3389/fpubh.2021.752481 (PMC8488164; doi:10.3389/fpubh.2021.752481)
Supplement: Supplementary file 1 [file Table_1.pdf]

Appendix

List of sectors in the GTAP 10 Database:

	Code	Description
1	pdr	Rice: seed, paddy (not husked)
2	wht	Wheat: seed, other
3	gro	Other Grains: maise (corn), sorghum, barley, rye, oats, millets, other cereals
4	v_f	Veg & Fruit: vegetables, fruit and nuts, edible roots and tubers, pulses
5	osd	Oil Seeds: oil seeds and oleaginous fruit
6	c_b	Cane & Beet: sugar crops
7	pfb	Fibres crops
8	ocr	Other Crops: stimulant; spice and aromatic crops; forage products; plants and parts of plants used primarily in perfumery, pharmacy, or for insecticidal, fungicidal or similar purposes; beet seeds (excluding sugar beet seeds) and seeds of forage plants; natural rubber in primary forms or in plates, sheets or strip, living plants; cut flowers and flower buds; flower seeds, unmanufactured tobacco; other raw vegetable materials nec
9	ctl	Cattle: bovine animals, live, other ruminants, horses and other equines, bovine semen
10	oap	Other Animal Products: swine; poultry; other live animals; eggs of hens or other birds in shell, fresh; reproductive materials of animals; natural honey; snails, fresh, chilled, frozen, dried, salted or in brine, except sea snails; edible products of animal origin n.e.c.; hides, skins and furskins, raw; insect waxes and spermaceti, whether or not refined or coloured
11	rmk	Raw milk
12	wol	Wool: wool, silk, and other raw animal materials used in textile
13	frs	Forestry: forestry, logging and related service activities
14	fsh	Fishing: hunting, trapping and game propagation including related service activities, fishing, fish farms; service activities incidental to fishing
15	coa	Coal: mining and agglomeration of hard coal, lignite and peat
16	oil	Oil: extraction of crude petroleum, service activities incidental to oil and gas extraction excluding surveying (part)
17	gas	Gas: extraction of natural gas, service activities incidental to oil and gas extraction excluding surveying (part)
18	oxt	Other Mining Extraction (formerly omn): mining of metal ores; other mining and quarrying
19	cmt	Cattle Meat: fresh or chilled; meat of buffalo, fresh or chilled; meat of sheep, fresh or chilled; meat of goat, fresh or chilled; meat of camels and camelids, fresh or chilled; meat of horses and other equines, fresh or chilled; other meat of mammals, fresh or chilled; meat of mammals, frozen; edible offal of mammals, fresh, chilled or frozen
20	omt	Other Meat: meat of pigs, fresh or chilled; meat of rabbits and hares, fresh or chilled; meat of poultry, fresh or chilled; meat of poultry, frozen; edible offal of poultry, fresh, chilled or frozen; other meat and edible offal, fresh, chilled or frozen; preserves and preparations of meat, meat offal or blood; flours, meals and pellets of meat or meat offal, inedible; greaves

21	vol	Vegetable Oils: margarine and similar preparations; cotton linters; oil-cake and other residues resulting from the extraction of vegetable fats or oils; flours and meals of oil seeds or oleaginous fruits, except those of mustard; vegetable waxes, except triglycerides; degras; residues resulting from the treatment of fatty substances or animal or vegetable waxes; animal fats
22	mil	Milk: dairy products
23	pcr	Processed Rice: semi- or wholly milled, or husked
24	sgr	Sugar and molasses
25	ofd	Other Food: prepared and preserved fish, crustaceans, molluscs and other aquatic invertebrates; prepared and preserved vegetables, pulses and potatoes; prepared and preserved fruits and nuts; wheat and meslin flour; other cereal flours; groats, meal and pellets of wheat and other cereals; other cereal grain products (including corn flakes); other vegetable flours and meals; mixes and doughs for the preparation of bakers' wares; starches and starch products; sugars and sugar syrups n.e.c.; preparations used in animal feeding; lucerne (alfalfa) meal and pellets; bakery products; cocoa, chocolate and sugar confectionery; macaroni, noodles, couscous and similar farinaceous products; food products n.e.c.
26	b_t	Beverages and Tobacco products
27	tex	Manufacture of textiles
28	wap	Manufacture of wearing apparel
29	lea	Manufacture of leather and related products
30	lum	Lumber: manufacture of wood and of products of wood and cork, except furniture; manufacture of articles of straw and plaiting materials
31	ppp	Paper & Paper Products: includes printing and reproduction of recorded media
32	p_c	Petroleum & Coke: manufacture of coke and refined petroleum products
33	chm	Manufacture of chemicals and chemical products
34	bph	Manufacture of pharmaceuticals, medicinal chemical and botanical products
35	rpp	Manufacture of rubber and plastics products
36	nmm	Manufacture of other non-metallic mineral products
37	i_s	Iron & Steel: basic production and casting
38	nfm	Non-Ferrous Metals: production and casting of copper, aluminium, zinc, lead, gold, and silver
39	fmp	Manufacture of fabricated metal products, except machinery and equipment
40	ele	Manufacture of computer, electronic and optical products
41	eeq	Manufacture of electrical equipment
42	ome	Manufacture of machinery and equipment n.e.c.
43	mvh	Manufacture of motor vehicles, trailers and semi-trailers
44	otn	Manufacture of other transport equipment
45	omf	Other Manufacturing: includes furniture
46	ely	Electricity; steam and air conditioning supply
47	gdt	Gas manufacture, distribution
48	wtr	Water supply; sewerage, waste management and remediation activities
49	cns	Construction: building houses factories offices and roads
50	trd	Wholesale and retail trade; repair of motor vehicles and motorcycles
51	afs	Accommodation, Food and service activities
52	otp	Land transport and transport via pipelines

53	wtp	Water transport
54	atp	Air transport
55	whs	Warehousing and support activities
56	cmn	Information and communication
57	ofi	Other Financial Intermediation: includes auxiliary activities but not insurance and pension funding
58	ins	Insurance (formerly isr): includes pension funding, except compulsory social security
59	rsa	Real estate activities
60	obs	Other Business Services nec
61	ros	Recreation & Other Services: recreational, cultural and sporting activities, other service activities; private households with employed persons (servants)
62	osg	Other Services (Government): public administration and defense; compulsory social security, activities of membership organisations n.e.c., extra-territorial organisations and bodies
63	edu	Education
64	hht	Human health and social work
65	dwe	Dwellings: ownership of dwellings (imputed rents of houses occupied by owners)
